# Feature Extraction Using Sparse Kernel Non-Negative Matrix Factorization for Rolling Element Bearing Diagnosis

**DOI:** 10.3390/s21113680

**Published:** 2021-05-25

**Authors:** Lin Liang, Xingyun Ding, Fei Liu, Yuanming Chen, Haobin Wen

**Affiliations:** 1 School of Mechanical Engineering, Xi’an Jiaotong University, Xi’an 710049, China; lianglin@xjtu.edu.cn (L.L.); d1519082309@stu.xjtu.edu.cn (X.D.); chenyuanming1992@stu.xjtu.edu.cn (Y.C.); wenhaobin@stu.xjtu.edu.cn (H.W.); 2 Key Laboratory of Education Ministry for Modern Design and Rotor-Bearing System, Xi’an Jiaotong University, Xi’an 710049, China

**Keywords:** sparse kernel nonnegative matrix factorization, time–frequency distribution, rolling element bearing, feature extraction

## Abstract

For early fault detection of a bearing, the localized defect generally brings a complex vibration signal, so it is difficult to detect the periodic transient characteristics from the signal spectrum using conventional bearing fault diagnosis methods. Therefore, many matrix analysis technologies, such as singular value decomposition (SVD) and reweighted SVD (RSVD), were proposed recently to solve this problem. However, such technologies also face failure in bearing fault detection due to the poor interpretability of the obtained eigenvector. Non-negative Matrix Factorization (NMF), as a part-based representation algorithm, can extract low-rank basis spaces with natural sparsity from the time–frequency representation. It performs excellent interpretability of the factor matrices due to its non-negative constraints. By this virtue, NMF can extract the fault feature by separating the frequency bands of resonance regions from the amplitude spectrogram automatically. In this paper, a new feature extraction method based on sparse kernel NMF (KNMF) was proposed to extract the fault features from the amplitude spectrogram in greater depth. By decomposing the amplitude spectrogram using the kernel-based NMF model with L1 regularization, sparser spectral bases can be obtained. Using KNMF with the linear kernel function, the time–frequency distribution of the vibration signal can be decomposed into a subspace with different frequency bands. Thus, we can extract the fault features, a series of periodic impulses, from the decomposed subspace according to the sparse frequency bands in the spectral bases. As a result, the proposed method shows a very high performance in extracting fault features, which is verified by experimental investigations and benchmarked by the Fast Kurtogram, SVD and NMF-based methods.

## 1. Introduction

As the rotary support component of most machinery, the fault detection and diagnosis of the rolling element bearing is crucial to prevent machinery breakdowns [1]. A variety of bearing fault detection techniques such as acoustic emission, electrostatic and vibration are used meticulously by industrial enterprises [2,3,4,5]. Among them, vibration monitoring is the most established diagnostic technique for rolling element bearing.

While a localized defect occurs on the surface of a relatively moving part, a series of impulses will be excited periodically or quasi-periodically. Due to different natural frequencies for rotating structure parts, multiple resonance regions reflecting impulse information will exist in the vibration signal of a mechanical system [6]. Inevitably, in the early stage of bearing failure, the noise energy usually contaminates the fault information of resonance regions.

Generally, an envelope-based analysis is one of the most popular technologies for bearing fault detection, because the envelope demodulation of a vibration signal can provide more valuable information [7,8,9]. Frequency band selection is substantial to get a successful envelope demodulation, because fault features cannot be extracted if the frequency band is not selected appropriately. Therefore, two algorithms were proposed, based on STFT and wavelet packet transform, respectively, to generate the Kurtogram and obtain the optimal frequency band with maximum kurtosis [10,11]. Apart from Kurtogram, another representative method is the Protugram [12], where the maximum kurtosis is extracted from the envelope signal instead of the raw signal. The Autogram [13] selects the optimal band with the highest impulsiveness also based on the maximum kurtosis, but unlike the Kurtogram, it is calculated based on the unbiased autocorrelation of the squared envelope of the demodulated signals. The Sparsogram [14] is based on the sparsity level on different bands based on the wavelet packet, and the Infogram utilizes the negentropy as a feature to detect the impulsive bands of the signal for demodulation [15]. SVD, which can adaptively extract representative features from the time–frequency distribution or Hankel matrix for the bearing fault diagnosis [16,17], has been proposed as a supplement to the envelope demodulation method in recent years. Jiang et al. utilized the ratio of singular values as an evaluation for the fault feature and further introduced the difference spectrum to select the informative singular vector due to different singular values for the signal and noise [18]. Xu et al. chose the anti-averaging method for sub-signal reconstruction and combined the SVD and squared envelope spectrum to identify the fault type. By the way, the wavelet transform matrix [19] can also be used for the input matrix of the SVD. It is worth noting that the SVD-based method is essentially the maximum variance projection, which means that the components with higher energy will be decomposed first, while the fault information is always neglected. To unravel this problem, an algorithm called RSVD was proposed based on the periodicity of the defect characteristics [20]. Mathematically, SVD-based methods only impose orthogonal constraints, so the value of the eigenvector atoms can be positive or negative. Such eigenvectors are usually associated with multiple frequency components, which will make the decomposition hard to understand and induce noise in the final results.

NMF has been widely used in audio source separation because of its ability to automatically separate components with different frequency and time information in the spectrogram [21,22]. By this virtue, researchers introduced NMF to the field of bearing fault detection to find the part-based representations of the bearing fault signal. Different from SVD, NMF imposes a non-negativity constraint on low-rank subspaces, so it has a better interpretability and can additively separate the components in frequency bands. For the amplitude spectrogram of the bearing fault signal, NMF can group the spectral components and sparsely present them in the factor matrices. Liang et al. [23] employed a deconvolution method for extracting impulse excitation using Convolution NMF and characterized the localized features of the impulse response, including the resonance frequency band and the attenuation response, effectively. Liu et al. [24] performed the Semi-NMF for the time–frequency matrix reconstruction, based on the vibration signal with a rolling element bearing defect, to extract the impulse response. Based on the amplitude spectrogram decomposition, Wodecki et al. [25] employed NMF to cluster the spectral components into the base matrix so that the optimal frequency band can be adaptively obtained. In essence, the above NMF-based developments were used to seek low-rank representations of high-dimensional time–frequency matrices for the bearing fault diagnosis based on a linear mixing model.

Considering that the kernel methods can offer a nonlinear mapping of the input space, some kernel-based NMF approaches [26,27,28] have been proposed to obtain a more trustworthy solution. Generally, as one of the outstanding properties, KNMF can provide a sparser representation of the data than NMF. Such properties can restrain the noise and help us select the frequency band more efficiently while we perform a fault diagnosis based on the amplitude spectrogram. Unfortunately, although the factor matrices obtained by KNMF are naturally sparse, it is difficult for users to control the sparse degree of representation. Hence, sparsity constraints can be imposed either with projections or L1 regularization on the KNMF model to induce a sparser solution.

We propose a novel feature extraction method in this paper, based on linear kernel NMF with a sparsity constraint, to detect fault characteristics from the amplitude spectrogram of bearing fault signal. To our knowledge, it is the first time a L1 regularization has been associated with a linear kernel NMF model, and it is the first time such models are being used in the context of a bearing fault diagnosis. With the linear kernel function, the time–frequency distribution is naturally decomposed into the superposition of subspaces by NMF. With the L1 regularization, a sparse KNMF model was established to obtain sparser factor matrices, which have energy only at the feature frequency bands presented in the input amplitude spectrogram. Thus, the optimal subspace corresponding to periodic impulses can be selected effectively by the sparse frequency band distribution of the sparse factor matrix. The proposed method outperforms the SVD method for its good interpretability and sparse decomposition results. Different from the envelope-based methods, the proposed method takes the time–frequency matrix of the short-time Fourier transform (STFT) as the input and can limit the in-band noise that perplexes the envelope-based methods by sparse representation. Several engineering applications show that the proposed method performs better than the SVD and envelope-based methods, especially in early fault detection cases.

This paper is organized as follows: Section 2 provides the fundamental knowledge about KNMF. In Section 3, the principles of the multiplicative update rule of kernel NMF with sparsity constraint are shown in detail. Section 4 presents a feature extraction strategy based on sparse KNMF; the vibration signals of the rolling bearing faults are presented to evaluate the proposed method in Section 5. At last, the conclusions are drawn in Section 6.

## 2. Kernel Non-Negative Matrix Factorization

### 2.1. NMF

As one of the low-rank decomposition tools, the goal of NMF is to factor a non-negative matrix into the product of two non-negative matrices called the basis matrix and the coefficient matrix:

(1) $$X_{m\times n}\approx U_{m\times r}V_{r\times n}$$

Different from SVD, the non-negativity constraints are imposed on two factor matrices, which can effectively show the concept of part-based representation. To solve matrices ***U*** and ***V*** in Equation (1), the usual Frobenius norm-based cost function is as follows:

(2) $$\underset{U,V}{\min}\frac{1}{2}||X-UV|{|}_{F}^{2},\begin{array}{cc} s.t. & U,V \end{array}\geq 0$$

where ***U***(*m* × *r*) is defined as the basis matrix, ***V***(*r* × *n*) is a coefficient matrix, $||\cdot |{|}_{F}^{2}$ is the Frobenius norm and *r* << min(m,n). Considering Equation (2) is not convex to ***U*** and ***V*** simultaneously, besides the gradient algorithm, multiplicative and alternating least squares algorithms were also proposed.

### 2.2. Kernel NMF

With nonlinear mapping: X→φ(X). For the decomposition of X=UV, the factor matrix ***U*** can be defined by

(3) U=XW

where ***W*** is the transformation matrix, and each column of ***W*** satisfies the constraint that the sum is one. Thus, the cost function (2) can be rewritten as follows:

(4) $$\begin{array}{l} \min D_{F}(\varphi (X\|U_{\varphi }V))=\frac{1}{2}{\left\|\varphi (X)-\varphi (X)WV\right\|}_{F}^{2},\\ \begin{array}{cc} s.t. & W\geq 0,V\geq 0 \end{array} \end{array}$$

In theory, the kernel function can improve the sparsity of the factor matrices, but the sparsity is still influenced by the initialization, kernel function type and parameter value. We will show that, in these cases [29], the sparse constraints can effectively match the features of the data. Additionally, for the case of the spectrogram of the bearing vibration signals, it was hoped that the sparsity of the factor matrices would make the impact energy more concentrated, and it is easy to select the feature frequency band.

## 3. Sparse KNMF and Update Rule

Although several approaches have been proposed to effectively control the sparseness of ***U*** and ***V*** in references [30,31], the sparsity constraints may result in the loss of useful information [32]. To get better sparseness of the frequency information from the time–frequency distribution, the constraints about the *L*_1_ norm of the coefficient matrix ***V*** is utilized in KNMF, and the sparse kernel non-negative matrix factorization (SKNMF) is also proposed. Taking the sparseness constraints of ***V*** into account, the Lagrangian is given by

(5) $$\min L_{F}(W,V)=\frac{1}{2}{\left\|\varphi (X)-\varphi (X)WV\right\|}_{F}^{2}+\alpha {\left\|V\right\|}_{1}$$

where α is the constraint term parameter, and *λ* and *μ* are the Lagrange multipliers. Under the conditions of Karush-Kuhn-Tucker (KKT) optimality, (***W***, ***V***) is a stationary point of Equation (5) if

(6) $$\begin{array}{c} \begin{array}{cc} W\geq 0 & V\geq 0 \end{array},\\ \begin{array}{cc} {\left(\partial L_{F}(W,V)/\partial W\right)}_{ij}\geq 0 & {\left(\partial L_{F}(W,V)/\partial V\right)}_{ij}\geq 0 \end{array}\\ \begin{array}{cc} W_{ij}{\left(\partial L_{F}(W,V)/\partial W\right)}_{ij}=0 & V_{ij}{\left(\partial L_{F}(W,V)/\partial V\right)}_{ij}=0 \end{array} \end{array}$$

when $W_{ij}>0$ and $V_{ij}>0$, the two partial derivatives of the objective function, from Equation (6), are denoted as follows:

(7) $$\begin{array}{l} (\partial L(W,V))/\partial W=0\\ (\partial L(W,V))/\partial V=0 \end{array}$$

Further, the optimal solution of Equation (7) can be defined following function:

(8) $$\begin{array}{c} \varphi {\left(X\right)}^{T}\varphi (X)WVV^{T}-\varphi {\left(X\right)}^{T}\varphi (X)V^{T}-\lambda =0\\ W^{T}\varphi {\left(X\right)}^{T}\varphi (X)WV-W^{T}\varphi {\left(X\right)}^{T}\varphi (X)+\alpha \nabla_{V}{\left\|V\right\|}_{1}-\mu =0 \end{array}$$

where $\nabla_{V}{\left\|V\right\|}_{1}$ is the gradient vector and means of the partial derivative with respect to ${\left\|V\right\|}_{1}$. Obviously, because the *L*_1_ norm cannot be differentiated directly, the subdifferential method of the real value variable [33] is used for this problem.

Set $\nabla_{V}\left\|V\right\|={\left[\nabla_{v_{1}}{\left\|v_{1}\right\|}_{1},\nabla_{v_{2}}{\left\|v_{2}\right\|}_{2},\ldots ,\nabla_{vn}{\left\|v_{n}\right\|}_{n}\right]}^{T}$, where the *i*th element is defined as

(9) $$\nabla_{{\left(v_{n}\right)}_{i}}{\left\|v_{n}\right\|}_{1}=\frac{\partial {\left\|v_{n}\right\|}_{1}}{\partial {\left(v_{n}\right)}_{i}}=\{\begin{array}{c} \left\{+1\right\},{\left(v_{n}\right)}_{i}>0\\ {}[-1,+1],{\left(v_{n}\right)}_{i}=0\\ \left\{-1\right\},{\left(v_{n}\right)}_{i}<0 \end{array}$$

Due to the non-negative of the elements of matrix ***V***, the set of [+1, −1] is set to 0 to simplify the calculations, and the above equation can be rewritten as

(10) $$\nabla_{{\left(v_{n}\right)}_{i}}{\left\|v_{n}\right\|}_{1}=\frac{\partial {\left\|v_{n}\right\|}_{1}}{\partial {\left(v_{n}\right)}_{i}}=\{\begin{array}{c} \left\{+1\right\},{\left(v_{n}\right)}_{i}>0\\ \left\{0\right\},{\left(v_{n}\right)}_{i}=0 \end{array}$$

Let us define $K=\varphi {\left(X\right)}^{T}\varphi (X)$ and use the KKT optimality conditions. We can obtain the following update rules when *λ* and *μ* are set to 0:

(11) $$\begin{array}{l} W_{ij}=W_{ij}\frac{{\left(KV^{T}\right)}_{ij}}{{\left(KWVV^{T}\right)}_{ij}}\\ V_{ij}=V_{ij}\frac{{\left(W^{T}K\right)}_{ij}}{{\left(W^{T}KWV+\alpha \nabla_{V}{\left\|V\right\|}_{1}\right)}_{ij}} \end{array}$$

where the kernel matrix ***K*** can be obtained with kernel functions, such as the polynomial kernel functions, Gaussian kernel functions and so on.

## 4. Feature Extraction Strategy Based on SKNMF

**Figure 1:**
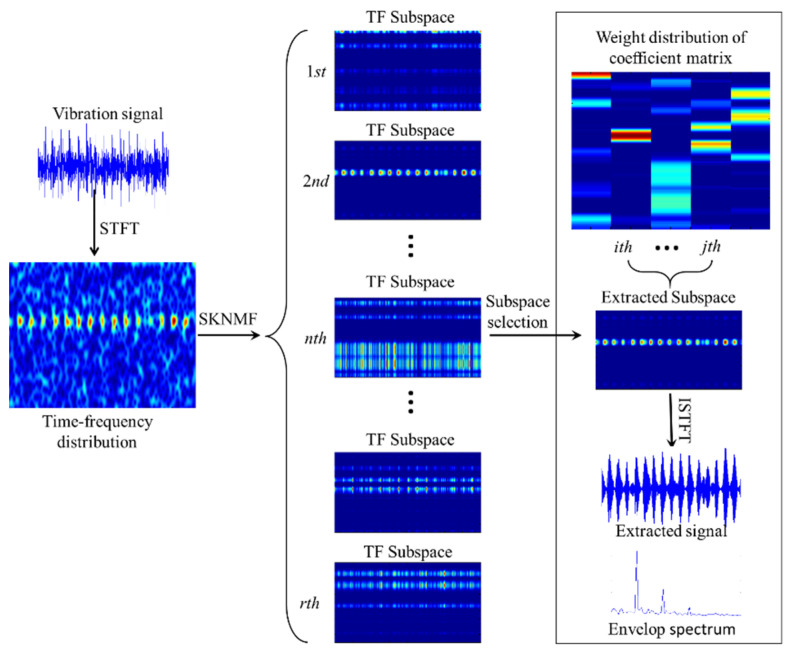
Scheme of the feature extraction based on the SKNMF.

For the bearing fault detection, the periodic impulses caused by a defect are localized at the resonance frequency band and corresponding time point in the time–frequency distribution. The fault impulse feature can be extracted by the advantages of SKNMF in the parts-based representation. The scheme of the whole strategy is displayed in Figure 1, and the implementation procedure is detailed as follows:

### 4.1. Time–frequency Distribution Construction

We denote by ***TF****_t,f_* the corresponding time–frequency matrix of the measured signal, and with STFT, ***TF****_t,f_* is described as below:

(12) $$TF_{t,f}=|\int_{-\infty }^{\infty }x(t+\tau )w(\tau )\exp(-2j\pi f\tau )d\tau |$$

where *x*(*t*) is the measured signal, *w*(*t*) is the window function (i.e., Hanning window) and *t* and *f* are the time and frequency, respectively.

### 4.2. Subspace Extraction with SKNMF

For the time–frequency space ***TF****_t,f_*, where its rows and columns represent the frequency and time location, respectively, the factor matrix ***V*** and transform matrix ***W*** are learned by the multiplicative updates of Equation (11). Then, in the feature space, according to Equations (1) and (3), we can obtain following expression:

(13) $$\varphi (TF_{t,f})=U_{\varphi }V$$

(14) $$U_{\varphi }=\varphi (TF_{t,f})W$$

where φ is mapping of the input space. $U_{\varphi }$ is the base matrix, where each column of $U_{\varphi }$ is denote by $u_{\varphi }{}_{k}$. Considering the generalization ability of the global kernel, the linear kernel function is selected and the kernel matrix $K=X^{T}X$. For ***TF****_t,f_*, we can obtain $\varphi (TF_{t,f})=TF_{t,f}$. Thus, according to Equation (13), the time–frequency space ***TF****_t,f_* is given by

(15) $$TF_{t,f}=\sum_{k=1}^{j}u_{\varphi }{}_{k}v_{k}$$

Naturally, Equation (15) shows that the time–frequency matrix ***TF****_t,f_* consists of a series of time–frequency subspace $TF_{t,f}^{k}=u_{\varphi }{}_{k}v_{k}$, and it can be rewritten by

(16) $$TF_{t,f}=\sum_{k=1}^{j}TF_{t,f}^{\left(k\right)}$$

### 4.3. Subspace Selection and Waveform Reconstruction

It is clear that each column of ***V*** has energy only at the main frequencies that are present in the subspace $TF_{t,f}^{\left(k\right)}$. We can interpret these columns as the frequency energy contained in the spectrogram. Due to the sparsity of ***V***, the corresponding frequencies in the subspace are more concentrated—namely, the noise interference components are less. Therefore, combining the resonance regions of the spectrum with the larger weight of ***V***, subspaces $TF_{t,f}^{\left(i\right)}(i\in (1,2,\ldots r))$ that represent the impulse features effectively can be determined. Then, using the inverse STFT to the estimated impulse components, the extracted signal $x_{s}$ can be obtained.

(17) $$x(t)=\frac{1}{2\pi }\int_{-\infty }^{\infty }\int_{-\infty }^{\infty }TF_{t,f}^{S}w(t-\tau )e^{jft}dfd\tau$$

where $TF_{t,f}^{S}=\sum TF_{t,f}^{\left(i\right)}{\left(i\in (1,2,\ldots r)\right)}^{T}$ is the selected time–frequency subspace.

### 4.4. Envelope Demodulation

Since the extracted signal $x_{s}$ contains the periodic impulse components, the envelope signal is obtained by $Z(t)=\sqrt{{\left|x_{s}(t)\right|}^{2}+{\left\{H[x_{s}(t)]\right\}}^{2}}$, where *H* is the Hilbert transform, and the frequency information of the periodic impulses can also be derived from the envelope spectrum of *Z(t)*.

## 5. Experimental Results

Two experiments were investigated to validate the proposed SKNMF, and the results are compared with not only the Kurtogram [10] and RSVD [20] but, also, NMF [34], sparse NMF (SNMF) [32] and kernel NMF (KNMF). Although a numerical experiment can be easily performed, it is difficult to accurately reflect the real working environment and complex frequency components of the vibration signal. Therefore, we used the real-world vibration data for comparison and analysis, while a measured data in practice was adopted for experiment verification.

### 5.1. Comparison Analysis Experiment

**Figure 2:**
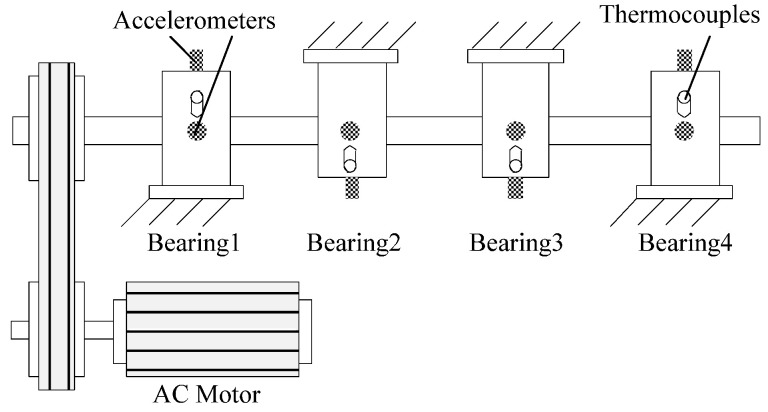
Bearing test rig.

The first experiment was designed to illustrate the efficiency of SKNMF for detecting an incipient bearing defect. The measured data came from the specially designed bearing test rig described in Figure 2. Four bearings were installed on a rotor shaft driven by an AC motor. The rotation speed was 2000 r/min, and acceleration sensors were mounted in the vertical and horizontal directions of each bearing seat. With a normal load and 20-kHz sampling frequency, the vibration data of the bearings was collected every 10 minutes. Finally, the outer race fault occurred in bearing 1 at the end of the experiment. For detailed information about the experiment, please refer to the literature [35].

**Figure 3:**
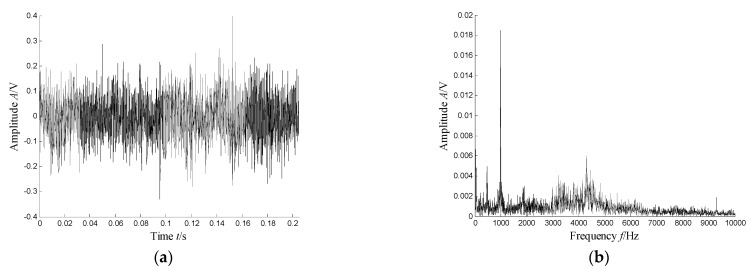
Waveform and spectrum of the outer race defect: (**a**) waveform and (**b**) spectrum.

According to the RMS value of the measured signals, we selected the vibration data collected during the 88.5 h for analysis, and the waveform and its spectrum are shown in Figure 3. Due to the early bearing defect, there are no periodic impulses shown in Figure 3a. For the spectrum in Figure 3b, not only the low frequency component is complicated, but also, different resonance regions can be observed.

**Figure 4:**
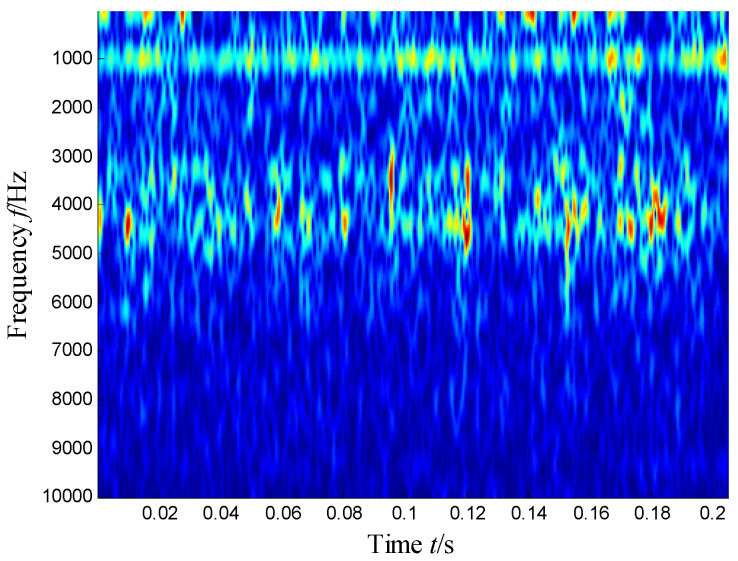
Time–frequency distribution of the outer race defect.

Figure 4 presents the time–frequency distribution obtained with the Hanning window of 20 samples. Obviously, the time–frequency space of the early fault signal presented a combination of time information and frequency energy. It was found that the frequency bands were focused at about 980 Hz, 1800 Hz, 3200 Hz and 4300 Hz. respectively, while the energy was lower for the frequency bands at about 1800 Hz and 3200 Hz.

**Figure 5:**
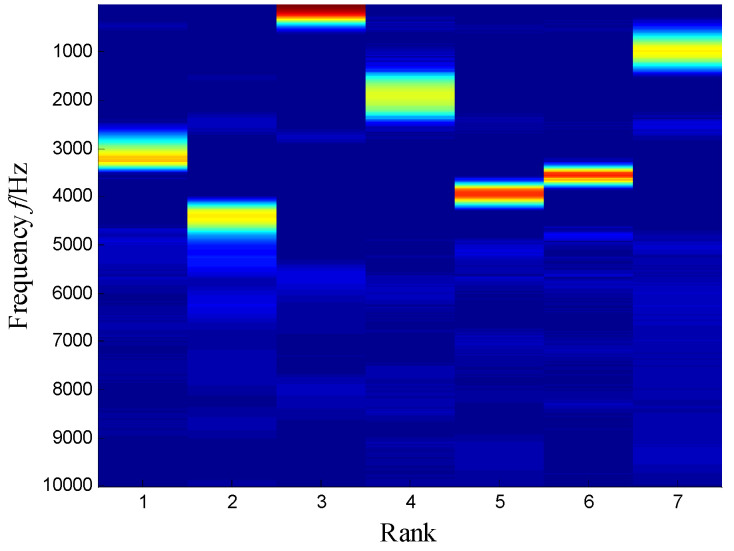
Coefficient matrix obtained by SKNMF.

**Figure 6:**
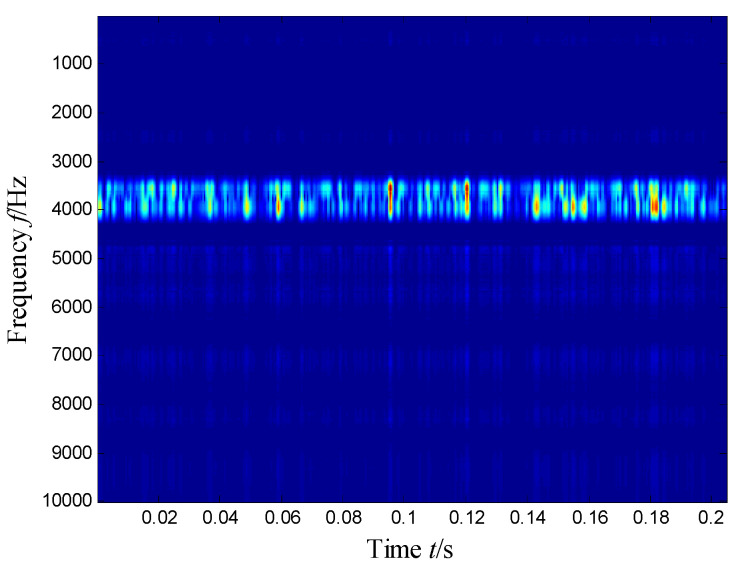
Selected time–frequency subspace.

**Figure 7:**
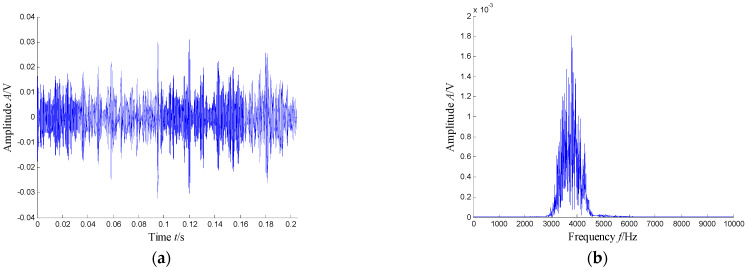
Waveform and spectrum of the extracted signal: (**a**) waveform and (**b**) spectrum.

**Figure 8:**
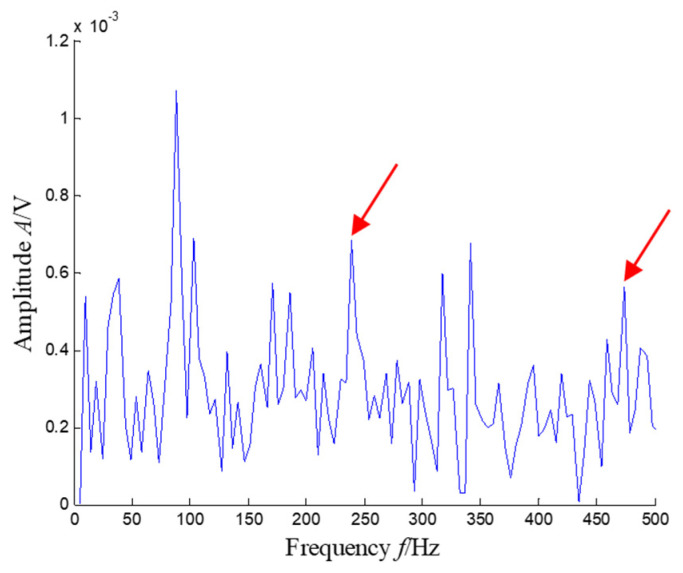
Envelope spectrum of the extracted signal (the first two order harmonics of *f*_BPFO_ are highlighted by two red arrows).

The SKNMF method was adopted to decompose the time–frequency space, where the elements in the initial matrices ***W*** and ***V*** were randomly chosen, and the factorization rank *r* and sparse coefficient *α* were set to 7 and 0.55, respectively. The coefficient matrix ***V*** is shown in Figure 5, where the vertical axis represents the frequency, and the horizontal axis is associated with a serial number of the subspace. It is shown that SKNMF with sparsity constraint tends to provide a sparse weight distribution in Figure 5, where the corresponding subspace has a more concentrate frequency band. From the frequency distribution of Figure 5, the corresponding weights of the fifth and sixth subspaces in the main frequencies were concentrated and larger. Therefore, the union of the two subspaces was selected, and the corresponding time–frequency subspace shown in Figure 6 was calculated by Equation (16). The extracted signal was obtained by inverse STFT, as shown in Figure 7a, and it revealed impulses compared with the original signal demonstrated in Figure 3. The spectrum of Figure 7b shows that the frequency band between 3100 Hz and 4400 Hz can be extracted adaptively, while the noise and other component interference can be eliminated effectively. The envelope analysis was performed on the extracted signal, and the corresponding envelope spectrum is shown in Figure 8. It is clear that the outer race fault characteristic *f*_BPFO_ (236.4 Hz) denoted by a red arrow is rather evident. Besides, the second order harmonic of *f*_BPFO_ denoted by another red arrow was also distinctive.

**Figure 9:**
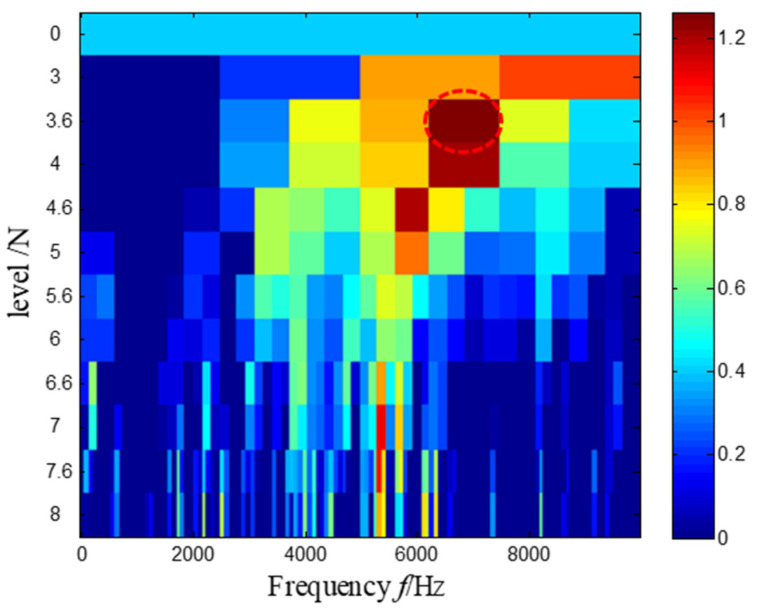
The Kurtogram and the optimal frequency band highlighted by a red dash circle.

**Figure 10:**
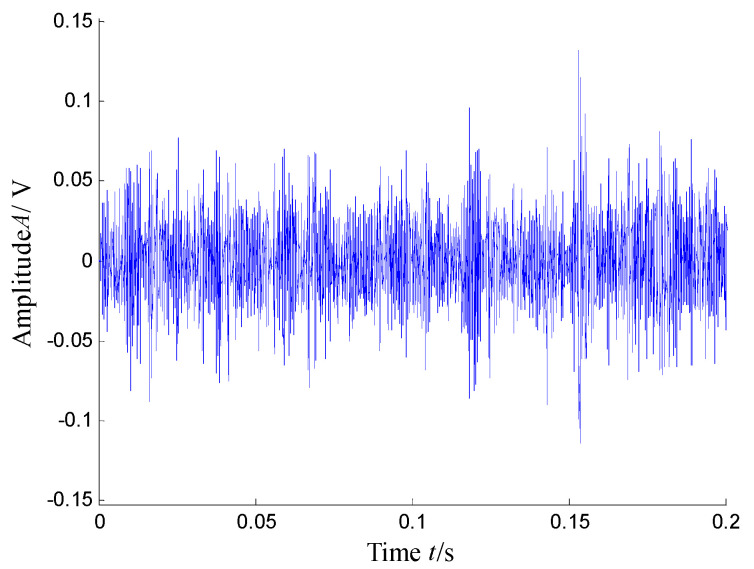
Filtered signal.

**Figure 11:**
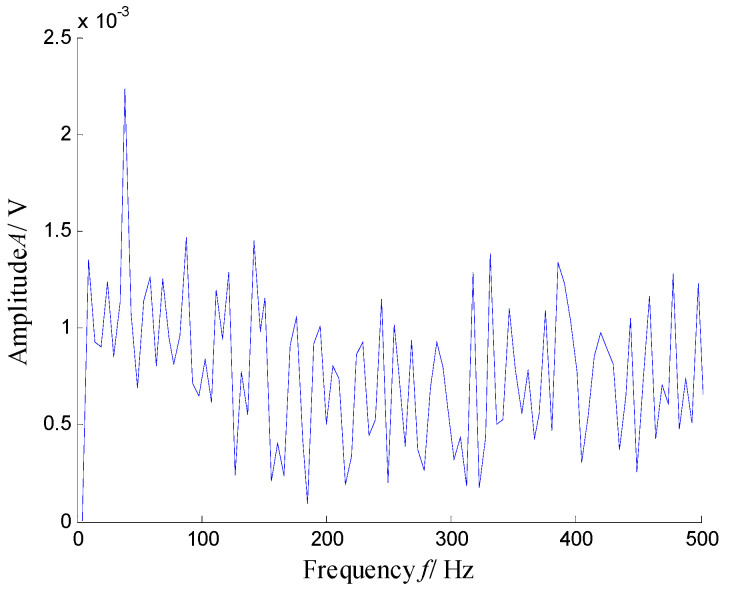
Envelope spectrum of the filtered signal.

For comparison, the Kurtogram [10] of the same measured signal is present in Figure 9, where it is found that the optimal demodulation band is from 6250 Hz to 7500 Hz. According to the obtained optimal demodulation band, a bandpass filter with a center frequency of 6875 Hz and a bandwidth of 1250 Hz is constructed and used to filter the raw vibration signal. The waveform of the filtered signal is presented in Figure 10. However, the results indicate that the filtered signal with maximum kurtosis still has noise interference, and the transient feature in the waveform is not very clear. When the envelope analysis is applied to this signal, little diagnostic information could be obtained, as shown in Figure 11. In fact, we know that kurtosis is a measure of the peakedness, so it easily tends to highlight the outliers of the signal caused by the noise.

**Figure 12:**
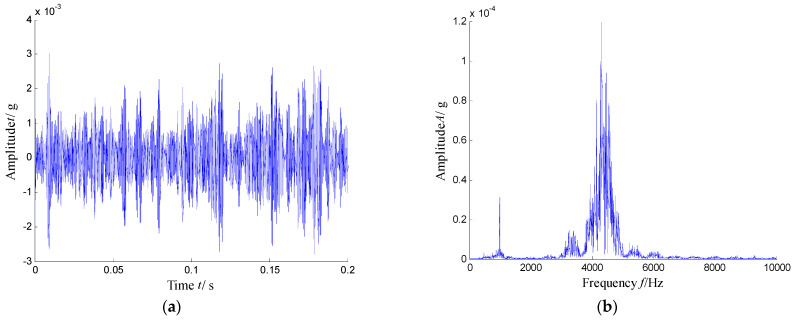
Waveform and spectrum of the 4th subspace obtained by the RSVD method: (**a**) waveform of the reconstructed signal and (**b**) spectrum of the reconstructed signal.

**Figure 13:**
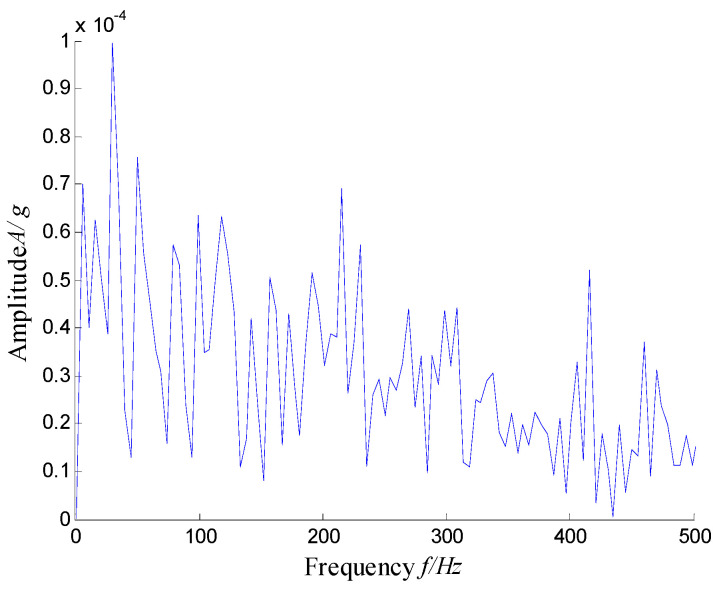
Envelope spectrum of the reconstructed signal obtained by the RSVD method.

The RSVD method [20] was then introduced to detect the impulse components. The waveform and spectrum of the reconstructed signal is shown in Figure 12, from which the fourth subspace was selected for construction according to the first 30 PMIs of the decomposed singular components. It is obvious that SVD can be decomposed by frequency information adaptively. However, compared with the above methods, except for the main frequency band, there are still other frequency components in Figure 12b. Therefore, from the envelope spectrum given in Figure 13, one can also find that the repetition frequency of the impulse is severely disturbed by the other interference components. In essence, only under orthogonality constraint, the decomposed eigenvector can be a positive or negative value. It means that the basis vector is related to many frequencies. Therefore, as the superposition of the subspace, the subspace usually includes not only multiple resonance regions but, also, noise.

**Figure 14:**
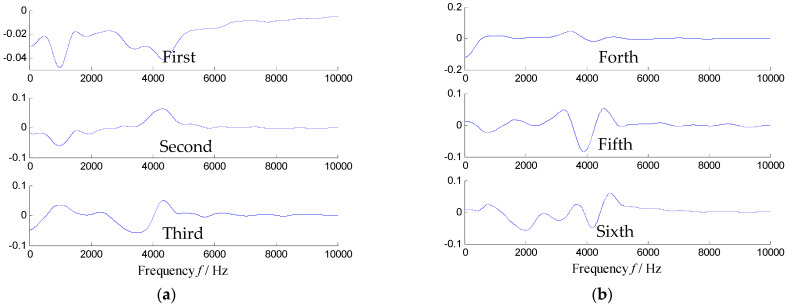
First six eigenvectors of Figure 4 extracted by SVD: (**a**) the first three eigenvectors and (**b**) the fourth–sixth eigenvectors.

To explain this drawback of the SVD method, the time–frequency matrix shown in Figure 4 is decomposed by SVD, where the decomposition equation is $TF_{t,f}^{}=USV$, and the column vectors of ***U*** and ***V*** are the orthonormal eigenvectors. Figure 14 illustrates the first six columns of ***V***, and it is well-known that the eigenvector of ***V*** reflects the frequency distribution of the raw signal. Since the SVD methods allow the atoms of the eigenvector as the negative value, as a result, each eigenvector mostly has positive and negative atoms in the main frequencies (i.e., 980 Hz, 1800 Hz, 3200 Hz, 4300 Hz and so on). Therefore, the subspace constructed by the eigenvectors of ***U*** and ***V*** also includes multiple frequency bands, which means that the noise cannot be limited effectively.

**Figure 15:**
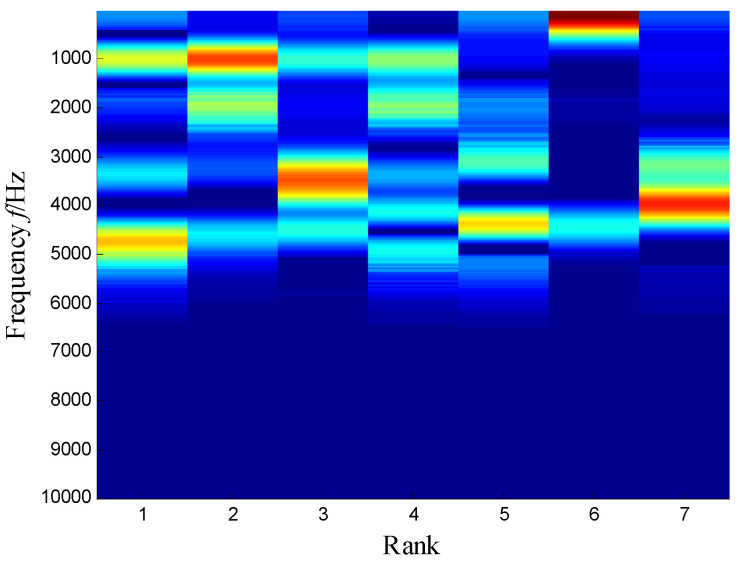
Coefficient matrix obtained by NMF.

**Figure 16:**
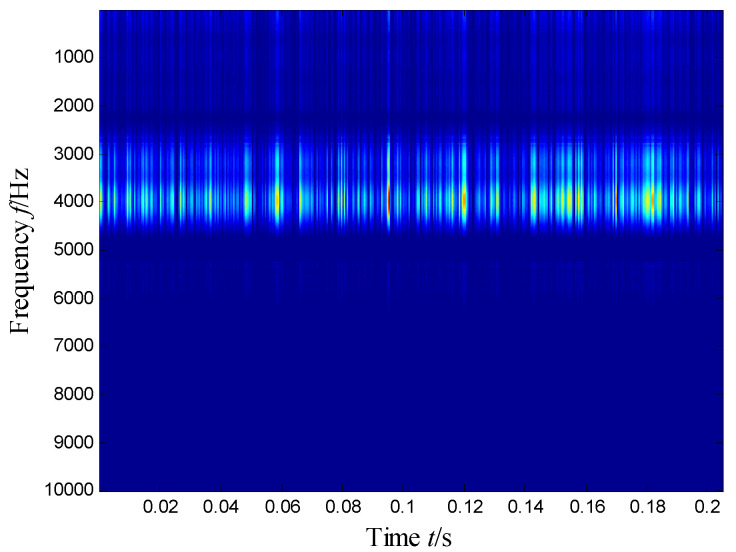
Selected time–frequency subspace for NMF.

**Figure 17:**
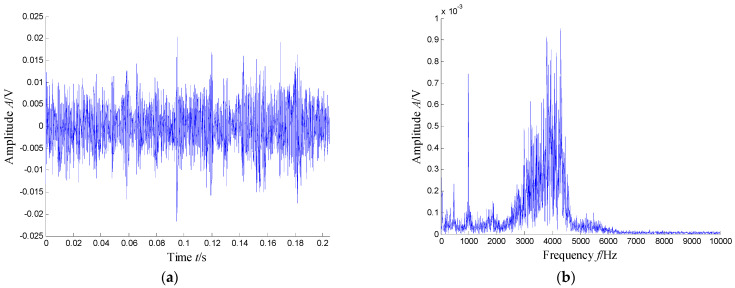
Waveform and spectrum of the extracted signal: (**a**) waveform of the extracted signal and (**b**) spectrum of the extracted signal.

**Figure 18:**
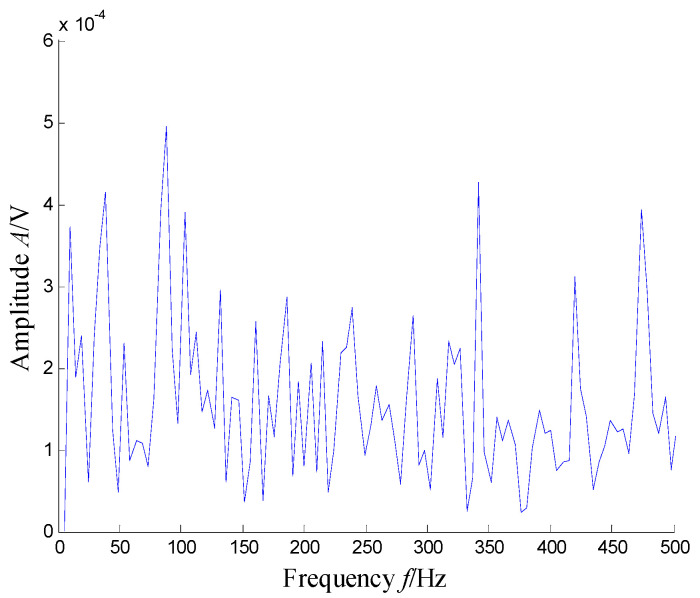
Envelope spectrum of the extracted signal.

To further test the effectiveness of the proposed method, Figure 15 illustrates the coefficient matrix ***V*** computed by NMF [34]. Certainly, one can easily see that the weights in KNMF are sparser than that of NMF. The poor sparsity makes each subspace factorized by NMF contain a lot of frequency components. Considering that the energy distribution of the seventh subspace is larger between 2400 Hz and 4700 Hz, the seventh subspace is selected as the extracted time–frequency subspace, as given in Figure 16. Using inverse STFT, the extracted waveform and spectrum are shown in Figure 17. Known from Figure 17b, except for the 986-Hz and 2400 Hz–4700-Hz frequency bands, the noise and other components are also observed. Thus, the periodic transient components were almost swallowed by noise in the waveform of Figure 17a. As a result, the envelope spectrum in Figure 18 shows that the fault characteristic *f*_BPFO_ is submerged by many high-amplitude frequency components.

SKNMF performs better than NMF, because the sparseness of the coefficient matrix is useful for the frequency information determination. It can be observed from Figure 5 that the sparsity of the decomposition result is strengthened, and SKNMF is sensitive to the different frequency band and can separate them effectively.

**Figure 19:**
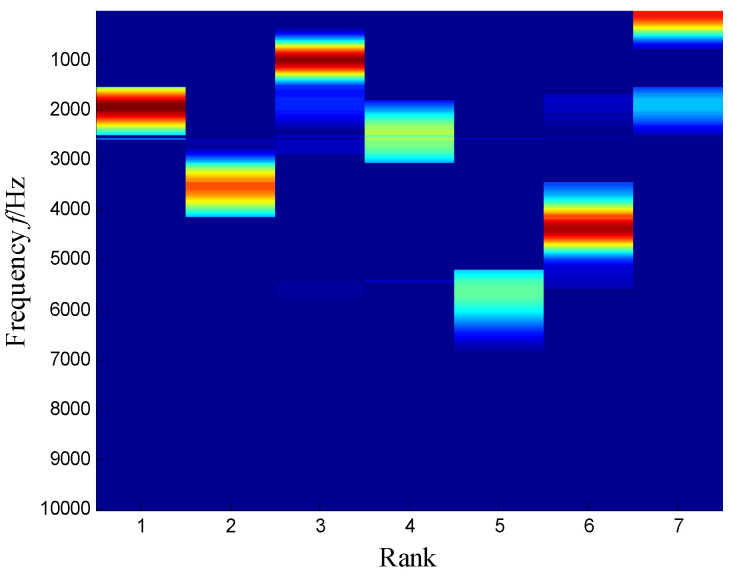
Coefficient matrix obtained by SNMF.

**Figure 20:**
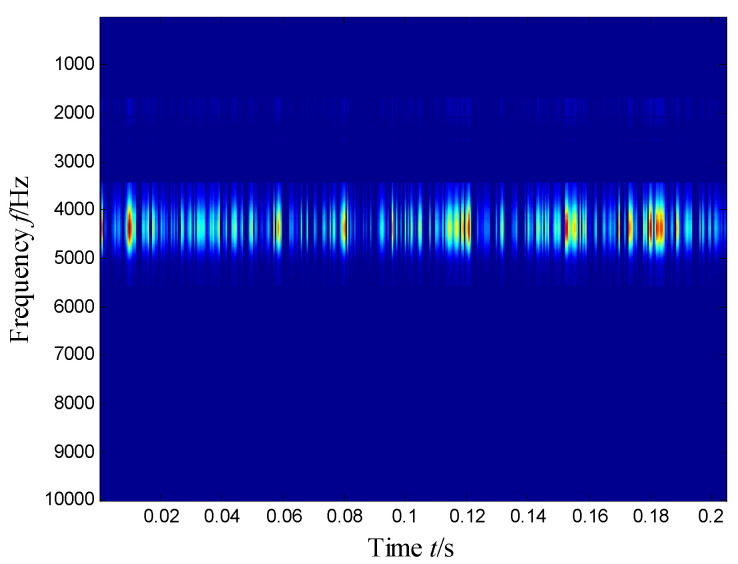
Selected time–frequency subspace for SNMF.

**Figure 21:**
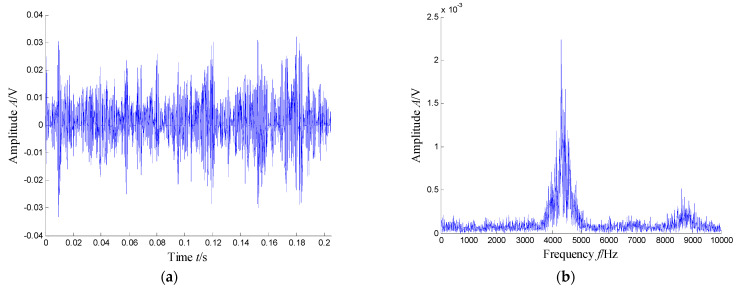
Waveform and spectrum of the extracted signal: (**a**) waveform and (**b**) spectrum.

**Figure 22:**
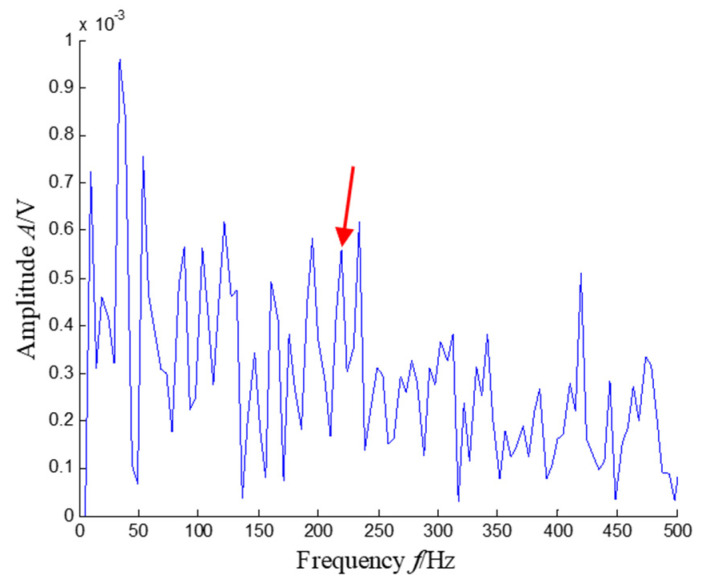
Envelope spectrum of the extracted signal (the fault characteristic *f*_BPFO_ is highlighted by a red arrow).

Figure 19 shows the coefficient matrix ***V*** of the sparse NMF method [32], where the rank *r* of the factorization is still set to 7, and parameters *η* and *β* are set to 0 and 0.55, respectively. Similar to the NMF, according to the spectrum and frequency distributions of the coefficient matrix of Figure 19, we chose the sixth subspace as the extracted time–frequency distribution. Thus, from the selected time–frequency subspace shown in Figure 20, one can obtain the extracted signal and spectrum. Compared with the original spectrum, the corresponding spectrum shown in Figure 21b includes not only 3600 Hz–5200-Hz frequency bands but, also, noise components. The transient signals in Figure 21a indicate that the extracted signal still has noise, and the transient feature is not very clear. Meanwhile, the fault frequency is also not easily distinguishable in the envelope spectrum, because only the first-order harmonic of *f*_BPFO_ is shown in Figure 22, where the fault character interfered with unknown high-amplitude components.

As the SNMF method was improved in the sparseness of the coefficient matrix, the extracted feature frequency band of the time–frequency distribution can be more concentrated. However, compared with SKNMF, except for the main frequency band, there were still other components that resulted in the feature frequency submerged in the envelope spectrum. Based on the kernel mapping and L1 norm-oriented sparseness, the SKNMF-based method can effectively eliminate the noise in the extracted signal. Therefore, the repetition frequency of the pulse characteristic can be highlighted.

**Figure 23:**
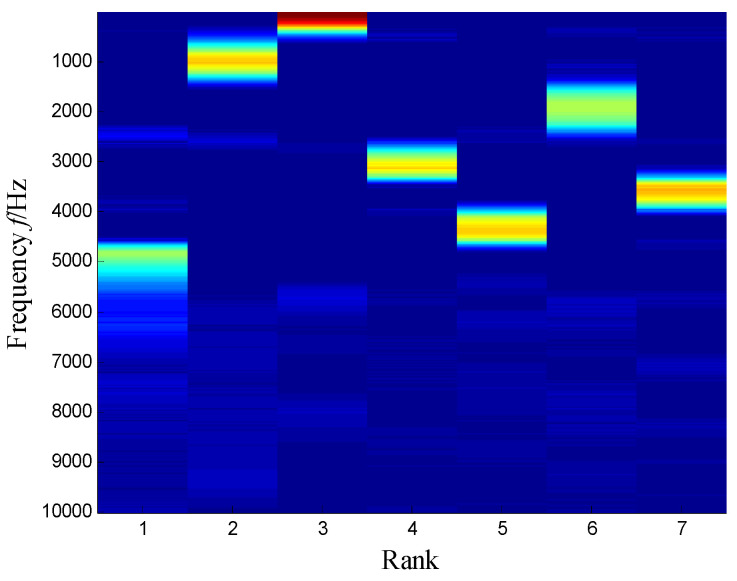
Coefficient matrix heat map obtained by KNMF.

Besides the above liner NMF-based models, the KNMF method with a multiplicative update strategy was employed to analyze the same signal. Figure 23 presents the heat map of coefficient matrix ***V***. Compared with the results of NMF, the decomposition results of KNMF had a certain sparsity, which was similar to the results of SNMF.

**Figure 24:**
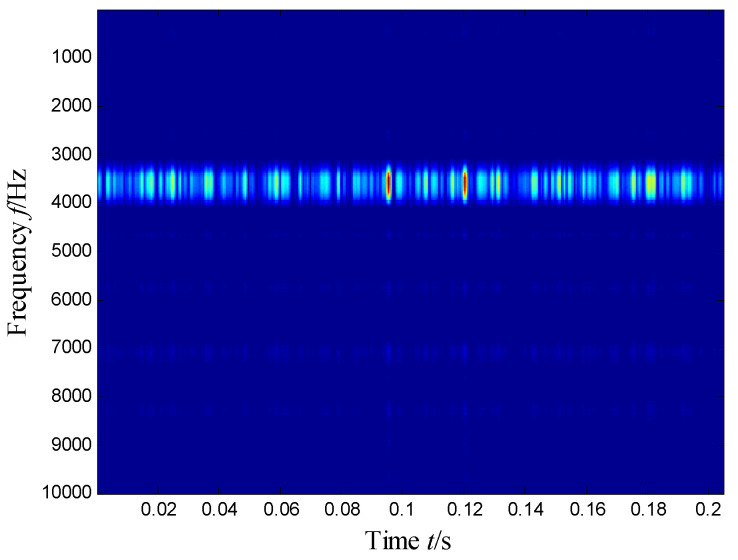
Selected time–frequency subspace for KNMF.

**Figure 25:**
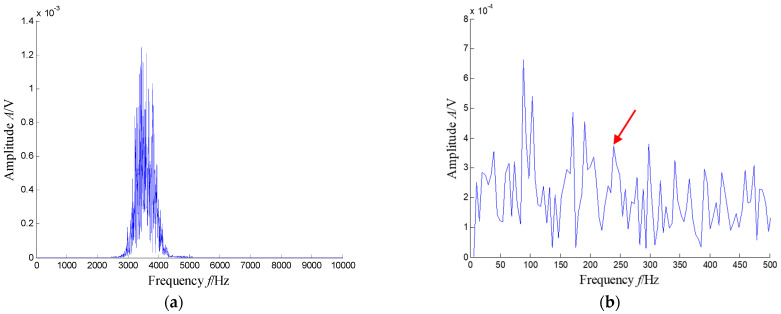
Spectrum and envelope spectrum of the extracted signal: (**a**) spectrum and (**b**) envelope spectrum (the fault characteristic *f*_BPFO_ is highlighted by a red arrow).

However, the sparseness of matrix ***V*** derived by KNMF was not as good as that of SKNMF, shown in Figure 5. Since the weight distribution of the subspace was not concentrated, a seventh subspace with the larger weight value was selected, and the extracted subspace is shown in Figure 24. Figure 25a,b shows the spectrum and the envelope spectrum of the extracted signal. Compared with Figure 7b, the extracted feature frequency bandwidth of KNMF was wider than that of SKNMF, which led to the defect feature KNMF extracted not being as clear as what SKNMF extracted.

### 5.2. Experimental Verification

#### 5.2.1. Test Rig

**Figure 26:**
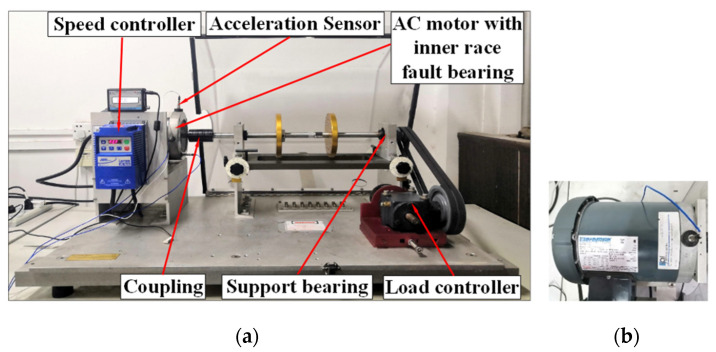
(**a**) Structure of the machinery fault simulator. (**b**) AC motor with acceleration sensor mounted.

A vibration signal, including multiple resonance regions, was also used to verify the SKNMF method in extracting the periodic impulses. The vibration data were obtained from the machinery fault simulator shown in Figure 26a, which was constructed with a motor, couplings, rotors, belt tensioning and gearbox. The rotating shaft was driven by an AC motor, and a fault simulation was conducted by replacing the fault motors. The motor bearing was SKF 6203. A constant load was applied by means of the magnetic brake. A zoomed version of the AC motor mounted with an acceleration sensor is shown in Figure 26b. The technical details of the acceleration sensor are listed in Table 1.

In the experiment, the AC motor with built-in inner race faulted bearings was installed under 1 pound of torque, and the PCB 352C68 ICP acceleration sensor was mounted on the bearing set of the motor. The rotary speed *f*_r_ controlled by a speed controller was 25 Hz, and the sample frequency was 10,240 Hz. According to the structural parameters of the bearing, the frequency of the inner race fault *f*_BPFI_ was 4.9*f*_r_ Hz.

**Figure 27:**
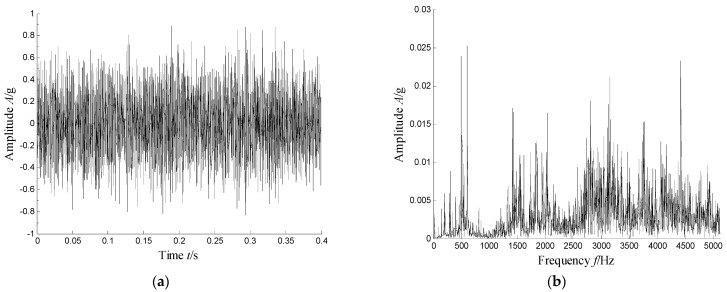
Waveform and spectrum of the vibration signal with an inner race fault bearing: (**a**) waveform and (**b**) spectrum.

**Figure 28:**
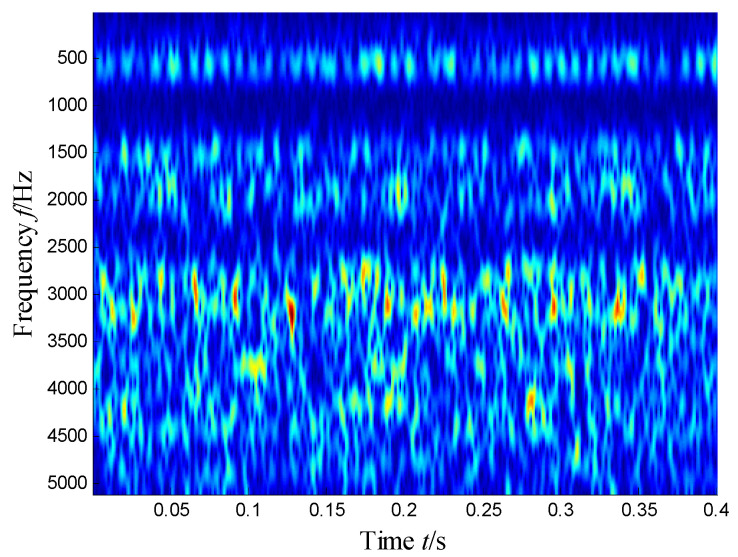
Time–frequency distribution of a defected bearing.

The waveforms and spectrum of the vibration signal of the defect bearing was plotted in Figure 27. Obviously, we can see that the many frequency bands were obviously visible. Obviously, the time–frequency distribution shown in Figure 28 presents the resonance region center focused at about 500 Hz, 2050 Hz and 3150 Hz and other high-frequency components.

#### 5.2.2. Results and Analysis

**Figure 29:**
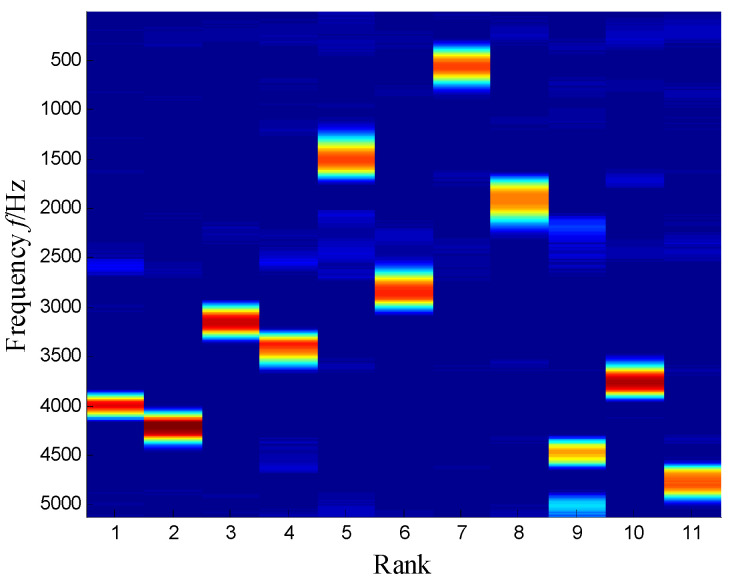
Coefficient matrix heat map of SKNMF.

**Figure 30:**
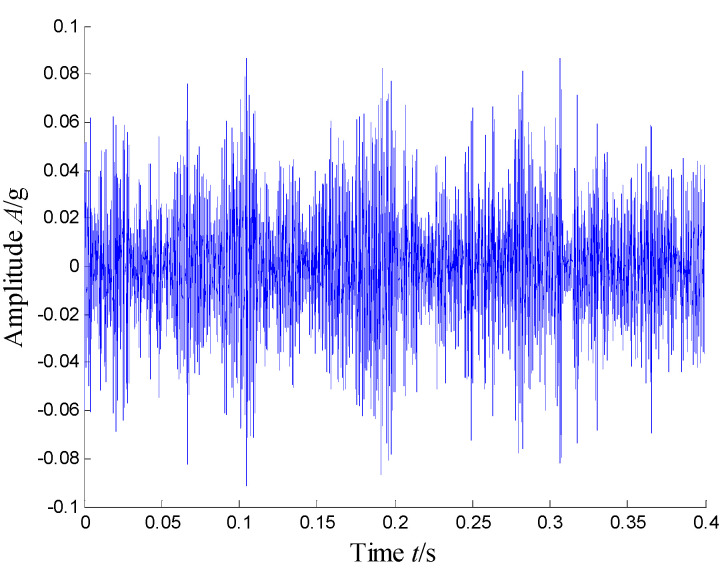
Extracted signal waveform.

**Figure 31:**
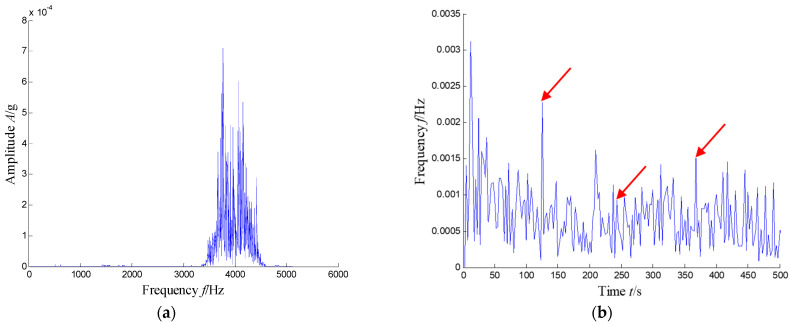
Spectrum and envelop spectrum of the extracted signal: (**a**) spectrum and (**b**) envelope spectrum (the first three order harmonics of *f*_BPFO_ are highlighted by three red arrows).

The proposed SKNMF-based method was adopted to decompose the time–frequency space, in which the rank *r* of the factorization was set to 11 because of more frequency bands. The extracted coefficient matrix is shown in Figure 29. From the weight distribution of Figure 29, the sparse weight of each rank was concentrated in different frequency bands, and the corresponding weight values in the subspaces of the first, second and tenth were larger. Therefore, the union of these subspaces was selected as the extracted signal given in Figure 30, and Figure 31 shows the spectrum and envelope spectrum of the extracted signal.

In Figure 31a, the feature frequency band between 3470 Hz and 4440 Hz can be extracted adaptively. The corresponding feature frequency *f*_BPFI_ that indicates the inner race defect can be observed clearly in Figure 31b, where the first three harmonics of *f*_BPFI_ are highlighted by three red arrows. It can be concluded from the results that the extracted signal with SKNMF can be used as an effective extraction means for the impulse feature caused by the localized defect.

**Figure 32:**
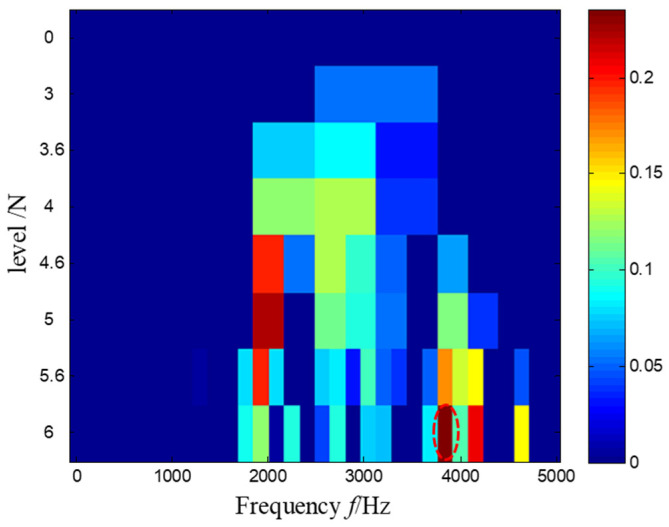
The Kurtogram and the optimal frequency band highlighted by a red dash circle.

**Figure 33:**
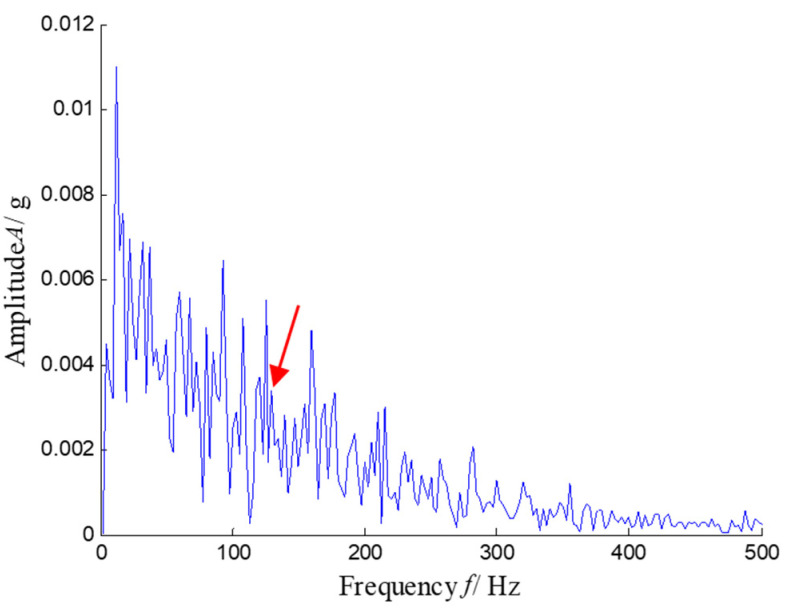
Envelope spectrum of the filtered signal.

Then, the Kurtogram was adapted to analyze the same measured signal in Figure 27. According to the maximum value principle shown in Figure 32, the envelope spectrum of the signal filtered by the optimal frequency band between 3760 Hz and 3920 Hz is demonstrated in Figure 33, where it is found that the repetition frequency of the impulses is not prominent.

There is no denying that the Kurtogram-based method has a certain ability to extract the feature frequency of the impulses. However, in Figure 33, the transient feature of the extracted signal was still disturbed by the noise and other frequencies, and the feature frequency in the envelope spectrum was less clear when compared with the envelope spectrum obtained by the proposed method, as shown in Figure 31b.

**Figure 34:**
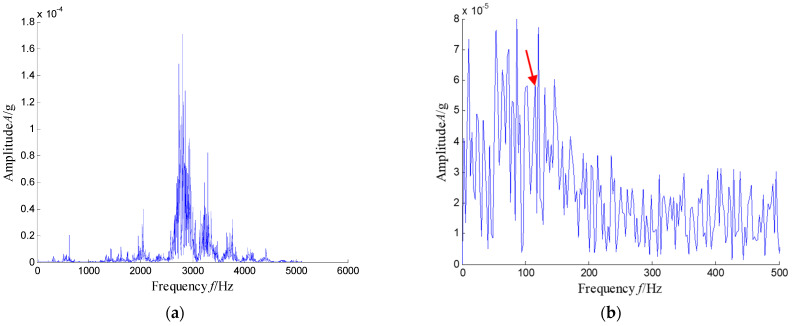
Spectrum and envelope spectrum of the 3rd subspace obtained by the RSVD method: (**a**) spectrum of the reconstructed signal and (**b**) envelope spectrum of the reconstructed signal.

The RSVD method was also adapted for comparison. By integrating 30 singular components, the spectrum and envelope spectrum of the reconstructed signal were obtained by the third subspace and illustrated in Figure 34, from which multiple frequency bands can be selected. The corresponding feature frequency of the inner race fault was not evidently displayed. Clearly, due to more frequency bands and noise contained in the vibration signal, for the adaptive decomposition of SVD, the feature signal and noise components were embedded in the same eigenvector subspace, which could affect the effect of the fault frequency extraction.

**Figure 35:**
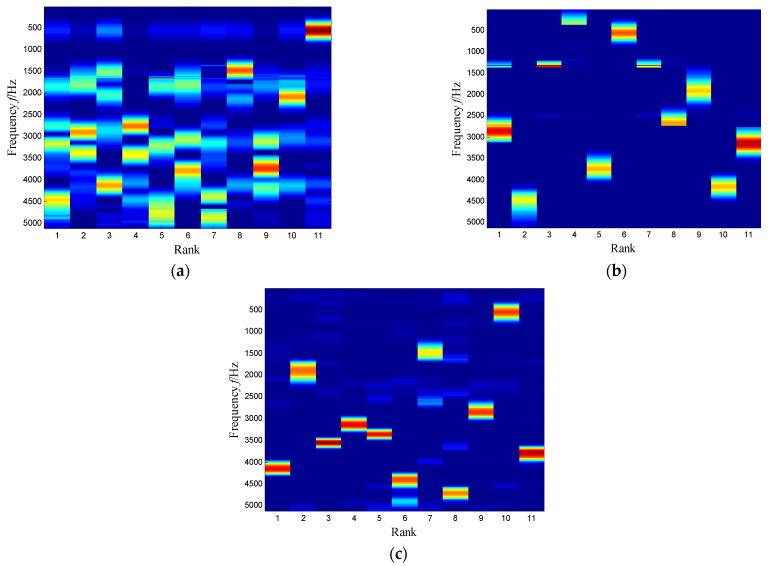
Coefficient matrixes with other NMF-based methods: (**a**) NMF-based, (**b**) SNMF-based and (**c**) KNMF-based.

In summary, SKNMF outperforms two excellent techniques. To clarify the essence of SKNMF, the coefficient matrices obtained by NMF, SNMF and KNMF are shown in Figure 35. It is obvious that the NMF-based method has the worst sparsity, while the factor matrix of KNMF is similar to SNMF because of its natural sparsity.

**Figure 36:**
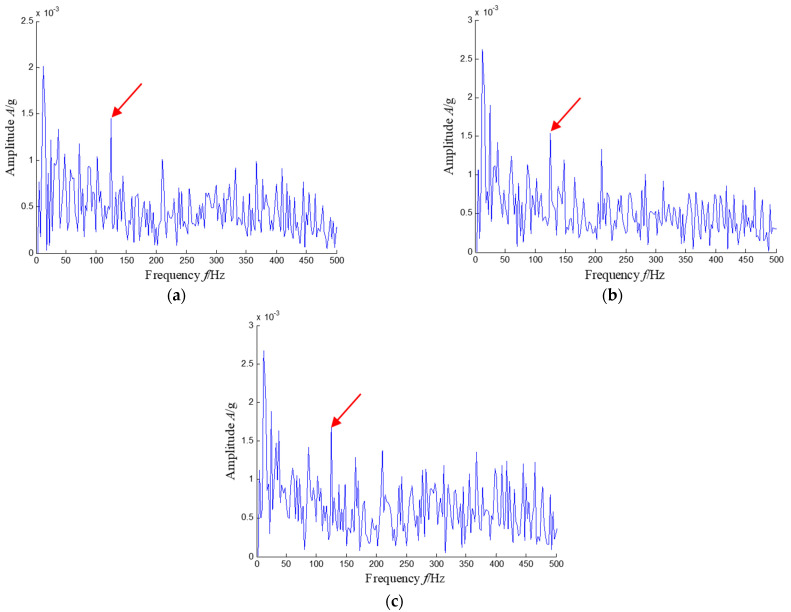
Envelope spectrums of the extracted signal with the other NMF-based methods. (**a**) NMF-based method, (**b**) SNMF-based method and (**c**) KNMF-based method (the fault characteristic *f*_BPFO_ is highlighted by a red arrow in each method).

Considering the distribution of subspace frequency band extracted by SKNMF, a similar frequency band was also selected in three NMF-based methods. The time–frequency subspace of NMF was selected with a union of the third, sixth and ninth subspaces. The envelope spectrum of the corresponding signal showed the fault-related signatures in Figure 36a. For the SNMF method, the union of the fifth and tenth subspaces was selected for the extracted result, and the envelope spectrum of the signal provided fault information in Figure 36b. Finally, KNMF was also adopted to process the weak signal and select the union of the first, third and tenth subspaces for analysis. The envelope spectrum of three extracted signals is plotted in Figure 36c. Although the envelope spectrum reflected the fault frequency, the signal-to-noise ratio was much lower than that of Figure 31b.

## 6. Conclusions

The vibration signal of a rolling bearing with a localized defect is generally a mixture of multiple components, in which the characteristic component, revealing the latent impulses, is often weak and difficult to detect. In this paper, a sparse kernel NMF model and corresponding feature extraction method were proposed to address this concern.

Two cases, one with an outer race fault and another with an inner race fault, were provided to illustrate that the proposed method can achieve the extraction of local weak signatures under the strong interference of a background noise. The proposed method was benchmarked by Fast Kurtogram, the SVD-based method, the NMF-based method, the SNMF-based method and the KNMF-based method. When the signal of interest was submerged by a strong background noise, the Fast Kurtogram showed how to keep the peak frequencies of interest below the noise level. The SVD-based method was confused by its orthogonal constraints, which often induce the eigenvectors associated with multiple frequency components, making the decomposition hard to understand and inducing noise in the final results. As for the NMF-based method, although it can extract the frequencies of interest, the final diagnosis result was not as clear as the proposed method. The comparison analysis experiment confirmed that SKNMF was superior to the other matrix decomposition methods, because it was sensitive to the different frequency bands and could separate them adaptively. As the result, the experimental verification allowed us to conclude that SKNMF is reliable enough to extract fault features and, hence, achieve early fault detection. It should be noted that the proposed method used an alternate iteration strategy, which made the algorithms not fast enough to be promoted for online monitoring.

## Figures and Tables

**Table 1:** Technical details of the acceleration sensor.

Sensor Model	Sensitivity	Measurement Range	Broadband Resolution	Frequency Range
PCB 352C68 ICP	(±10%) 100 mV/g (10.2 mV/(m/s²))	±50 g pk (±491 m/s² pk)	0.00016 g rms (0.0015 m/s² rms)	(±5%) 0.5 to 10,000 Hz
